# Clinical and molecular characteristics of Kabuki syndrome patients with missense variants—novel features and literature review

**DOI:** 10.3389/fgene.2024.1402531

**Published:** 2024-07-22

**Authors:** Snir Boniel, Maria Krajewska, Beata Pyrżak, Monika Paluchowska, Anna Majcher, Magdalena Zarlenga, Katarzyna Krenke, Robert Śmigiel, Anetta Jeziorek, Krystyna Szymańska, Krzysztof Szczałuba

**Affiliations:** ^1^ Department of Medical Genetics and Centre of Excellence for Rare and Undiagnosed Diseases, Medical University of Warsaw, Warsaw, Poland; ^2^ Department of Pediatrics and Endocrinology, Medical University of Warsaw, Warsaw, Poland; ^3^ Department of Neonatology, Medical University of Warsaw, Warsaw, Poland; ^4^ Department of Pediatric Pulmonology and Allergology, Medical University of Warsaw, Warsaw, Poland; ^5^ Department of Family and Pediatric Nursing, Medical University, Wroclaw, Poland; ^6^ Neurodiagnostic Unit, Medical University of Warsaw, Warsaw, Poland; ^7^ Department of Pediatric Neurology, Medical University of Warsaw, Warsaw, Poland

**Keywords:** Kabuki syndrome, missense variant, genotype, phenotype, behavior, development

## Abstract

Kabuki Syndrome (KS) encompasses a spectrum of clinical manifestations, primarily attributed to pathogenic variants in the *KMT2D* gene. This study aims to elucidate novel features in KS patients with missense variants, contrasting their presentation with both literature-reported cases of patients with missense pathogenic variants as well as other KS patients with truncating pathogenic variants. Employing a survey questionnaire and clinical evaluations, we examined ten KS patients with missense variants, focusing on their dysmorphism characteristics, behavior and psychomotor development. We identified unique features in missense variant patients, including foot hyperesthesia, musicality, and sensory integration disorders. Notably, despite similarities in developmental trajectories, distinct phenotypic traits emerged in missense variant cases, suggesting a potential genotype-phenotype correlation. These findings contribute to a deeper understanding of KS heterogeneity and underscore the importance of genotype-specific characterization for prognostic and therapeutic considerations. Further exploration of genotype-phenotype relationships promises to refine clinical management strategies and enhance patient outcomes in this complex syndrome.

## 1 Introduction

Kabuki Syndrome (KS, MIM#147920, MIM#300867, Orpha 2322) was first described in the early 1980s and was named after the characteristic facial features of actors in the Kabuki theater in Tokyo, Japan (Kuroki et al., 1981). Patients present with distinct facial dysmorphism (long palpebral fissures, lower eyelid eversion and a short columella with a depressed nasal tip), joint hyperlaxity, developmental delay, growth retardation and a wide spectrum of other manifestations.

*KMT2D* gene pathogenic variants have been identified as the main cause of KS. *KDM6A* gene pathogenic variants account for a minority of cases (Ng et al., 2010). Most *KDM6A* variants were characterized as either point mutations or microdeletions. The KMT2D protein is a histone (H3) lysine methyltransferase enzyme responsible for cell-type specific gene expression during differentiation (Bjornsson et al., 2014). *KDM6A* however encodes for a Histone (H3) lysine-27 demethylase. The KDM6A protein plays a key role in chromatin remodeling through a shared pathway and through interaction with the KMT2D protein in a SET1-like complex. It has been suggested to have a unique significance during embryonic life. *KMT2D* most likely plays a role in central nervous system, craniofacial, circulatory and bone development. Most *KMT2D* variants are protein truncating (nonsense/frameshift), while up to 30% are predicted to be missense (Cocciadiferro et al., 2018).

Missense *KMT2D* variants cause reduced histone methylation due to impaired WRAD protein complex formation (Cocciadiferro et al., 2018). They were identified along the entire length of the *KMT2D* gene. Using functional studies, it was found that some missense variants reduce histone methylation activity, thus confirming their character as predicted loss-of-function (Cocciadiferro et al., 2018). Individuals present with a wide spectrum of phenotypes and some express milder symptoms than those who carry protein-truncating *KMT2D* pathogenic variants. A novel KS phenotype caused by specific exon 38 or 39 missense *KMT2D* pathogenic variants was suggested (Cuvertino et al., 2020). It was originally described as a multiple malformation disorder distinct from KS. These patients consistently showed head and neck dysmorphism: choanal atresia, ear malformations, lacrimal system deformities, branchial cleft abnormality, neck pits, athelia or nipple hypoplasia (Cuvertino et al., 2020). Based on the suggestions from the recent reports we aim to further delineate Kabuki-Missense subtypes: one caused by specific exon 38 or 39 *KMT2D* missense variants and a second caused by an array of other *KMT2D* missense variants located elsewhere, most likely associated with a spectrum of milder phenotypes and dysmorphisms.

The original description of a specific multiple-malformation disorder linked to missense variants in exons 38 was recently refined (Tharreau et al., 2022). Two families with a history of autosomal dominant choanal atresia or nasolacrimal duct anomalies, thyroid dysfunction, hearing anomalies and nipple hypoplasia were described. The authors suggest that missense variants in exon 38 of *KMT2D* could lead to milder phenotypes. Additionally they detail syndactyly and polydactyly in patients with *KMT2D* missense variants, a finding that is rarely associated with KS (Tharreau et al., 2022).

Herein we present a case series of ten Kabuki Syndrome patients with confirmed *KMT2D*-missense pathogenic variants. One patient was diagnosed with a missense variant located in exon 39 and nine patients had confirmed missense variants located elsewhere, i.e., outside of exons 38 or 39. Patients’ novel features that broaden the phenotype of Kabuki-Missense are unique behaviors (e.g., musicality, elements of autism spectrum behavior), foot hyperesthesia and ear dysmorphism such as over-folded helix and protruding antitragus. Patients tended to present with a significantly milder facial dysmorphism than that described in truncating *KMT2D* pathogenic variants. Brachydactyly was not observed. We sum up the current knowledge about clinical phenotype associated with missense *KMT2D* variants.

## 2 Materials and methods

Ethical approval for our study was obtained from the Bioethics Committee of the Medical University of Warsaw (code: KB/144/2021). Informed consent for our study was obtained from all participating families as was permission to publish patient photographs. Ascertainment was driven by genotype in patients in whom *KMT2D* missense variants were identified by genetic tests either directed at *KMT2D* variants or next-generation sequencing tests. Patients (including 30 other KS patients with truncating pathogenic variants) were recruited from the Kabuki Syndrome Polska Group as well as from respective databases in the Departments of Medical Genetics at the Medical Universities of Warsaw and Wroclaw. Variants were classified according to the American College of Medical Genetics and Genomics (ACMG) guidelines (Richards et al., 2015). Four variants were classified as *Variants of Unknown Significance* (VUS), the remaining six as either likely pathogenic or pathogenic. All affected individuals underwent clinical phenotyping. All patients underwent a physical examination and a neurological examination. Anthropometric measurements were recorded. Data for statistical analysis was collected from the group of 9 KS patients with missense pathogenic variants and from the 30 KS patients with truncating pathogenic variants.

## 3 Clinical report

### 3.1 A patient with a *KMT2D* missense variant in exon 39

A male infant was born to young, healthy, and nonconsanguineous parents of two other healthy children. The pregnancy was complicated by intrauterine growth restriction. Increased nuchal translucency was confirmed upon ultrasonography. Karyotype from amniocytes was normal: 46, XY.

The patient was born spontaneously at the gestational age of 39 weeks with a birth weight of 2470g (<3rd WHO centile, -2SD (Ruault et al., 2020)), a birth length of 52 cm (50–85th WHO centile, -1SD (Ruault et al., 2020)) and a head circumference of 34 cm (15–50th WHO centile,-2SD (Ruault et al., 2020)), with APGAR scores of 7-2-0-5-6 respectively at 1, 3, 5, 10, and 20 min of life. After delivery the newborn underwent cardiopulmonary resuscitation. He was transferred to the Neonatal Intensive Care Unit under mechanical ventilation and was diagnosed with respiratory failure and acute respiratory distress syndrome. Abnormalities found upon chest imaging are described in Supplementary Figure S1.

**FIGURE 1:**
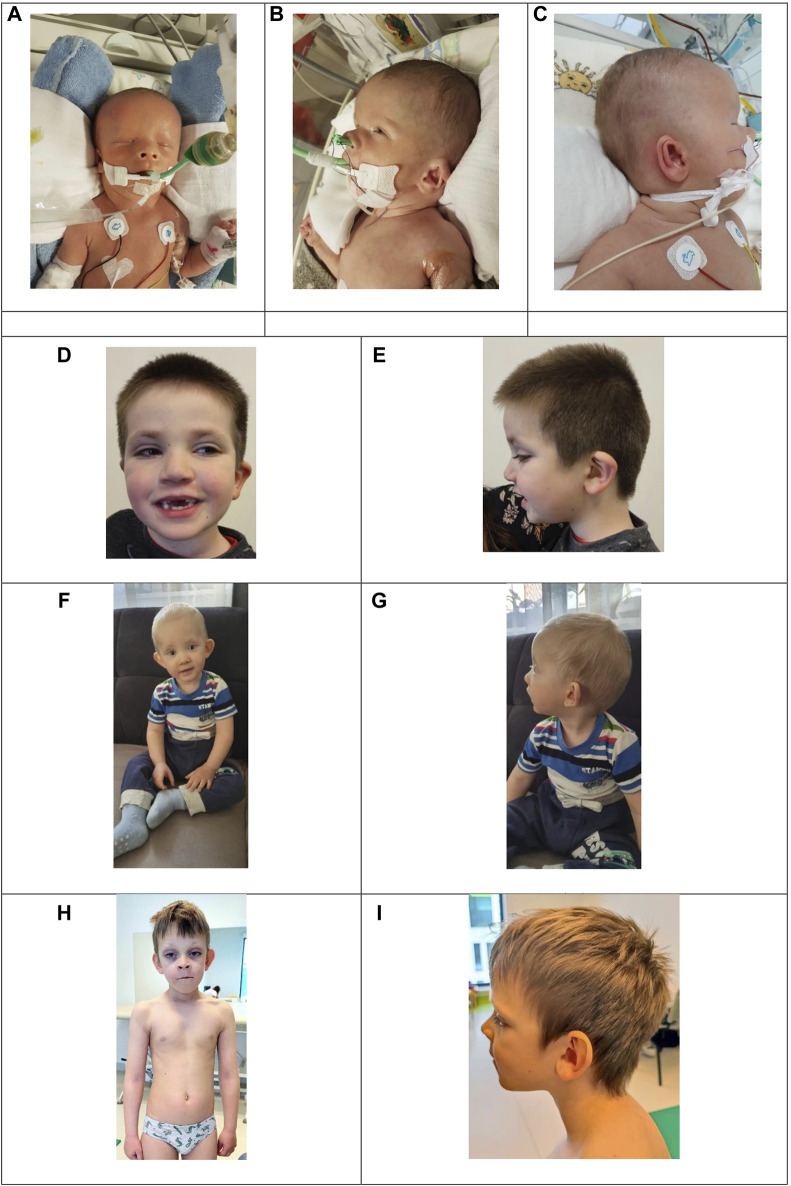
Images A, B, and C present dysmorphic features of the patient with a missense KMT2D variant in exon 39 c.10607G>C. **(A)** Broad forehead, hypertelorism, triangular nose with anteverted nares, athelia. **(B)** Overfolded upper helix, anteverted nares. **(C)** Outer ear dysmorphism: prominent antitragus and large earlobe. Images D to J show three patients with KMT2D missense variants outside of exons 38–39 presenting typical yet mild dysmorphic features. **(D)** and **(E)**: Patient 8 with a variant in exon 43, **(C)** 13961A>G, presenting long palpebral fissures, sparse eyebrows, epicanthal folds, lower eyelid eversion and abnormal dentition. **(F)** and **(G)**: Patient 7 with a variant in exon 49, **(C)** 15142C>T, presenting noticeable coloboma, lower eyelid eversion and prominent ears. **(H)** and **(I)**: Patient 4 with a variant in exon 49, **(C)** 15544G>C presenting long palpebral fissures, lower eyelid eversion, sparse eyebrows, prominent ears and kyphoscoliosis.

Physical examination revealed a broad forehead, microphthalmia, hypertelorism, low-set ears with an auricular dysmorphism, an overfolded upper helix, a protruding antitragus and a large earlobe; choanal atresia with anteverted nares and a triangular nose, athelia, hypospadias, and anal atresia (Figure 1). Anal atresia was surgically corrected by colostomy insertion on the 5th day of life.

Patent ductus arteriosus was diagnosed postnatally and closed spontaneously on the 4th day of life. Echocardiography confirmed its spontaneous reopening during the 5th week of life, and it was then reclosed surgically. Persistent pulmonary hypertension of the newborn was confirmed at birth and resolved upon inhalational nitric oxide therapy.

The child presented with several seizure episodes during the 11th day of life. Epileptic encephalopathy refractory to treatment was diagnosed. MRI T1-weighted imaging showed a hemorrhagic change in the internal capsule and cerebellum as well as lesions in areas of active myelinization. Bilateral defects of the inner ears, as defined by hypoplasia and dysplasia of the upper semicircular canals and aplasia of the posterior semicircular canals were confirmed. Neonatal Otoacoustic Emissions screening tests showed a bilateral lack of reaction. Upon neurological examination during the first weeks of life, evolution towards spastic tetraparesis was observed.

ArrayCGH results were normal. Due to an unclear clinical picture and a worsening general condition, Whole Exome Sequencing was performed. Cardiac arrest was confirmed during the 85th day of life. Bearing in mind the poor prognosis and severe brain damage traits upon imaging, as well as the exhaustion of diagnostic and therapeutic possibilities, resuscitation was not undertaken. The cause of death was severe cardiorespiratory failure due to severe bronchopulmonary dysplasia and other internal organ developmental abnormalities. Post-mortem Whole Exome Sequencing results showed the presence of a heterozygous *de novo* missense variant NM_003482.4, c.10607G>C (p. Arg3536Pro), chr12-49034200-C-G in exon 39 of the *KMT2D* gene, classified as VUS, PM2, PP2 according to ACMG criteria (Richards et al., 2015).

### 3.2 Patients with *KMT2D* missense variants outside of exons 38–39

We describe 9 patients - 2 females and 7 males. All patients’ genetic tests revealed *de novo* missense *KMT2D* pathogenic variants outside of exons 38–39, hypothesizing a separate phenotype. Detailed clinical characteristics and anthropometric measurements of all the nine individuals are shown in Supplementary Table S1. Below is the short summary of outstanding features.

All patients were born at term to nonconsanguineous healthy parents. Three patients experienced a childbirth complication: respiratory distress due to atypical pneumonia treated with antibiotic therapy and respiratory support. Cleft palate was observed in two individuals. A noticeable gross motor and fine motor delay was observed in all patients. Patients older than 3 years tended to express certain autism spectrum traits such as disproportionate emotionality (e.g., crying or screaming in a new environment) and a tendency to fixate their behaviors on one point (e.g., repetitive actions). Upon posture assessment, 4 patients exhibited prominent kyphoscoliosis. Upon neurological assessment, global hypotonia and a mild secondary balance disorder was evident in all patients.

Interestingly, all examined individuals showed foot hyperesthesia upon plantar reflex examination, a phenomenon which has not been described and associated with KS in the literature. Heightened sensitivity to sole touch stimulation in all of our patients was characterized by a jerk reaction, our patients retracted their feet bilaterally and refused to cooperate further with the examiner. Additionally patients 1, 3, 8, and 9 complained of foot pain upon touch stimulation, even uncontrollably crying as a response to this exam. A similar response was also observed upon any tactile plantar stimulation, as well as temperature stimulation and even while our patients were walking on a soft surface. That said, their gait did not seem to be affected by foot hyperesthesia while walking on a hard floor during examination.

All parents stated that their children had sensory integration disorder traits—sudden aggressive outbursts or tantrums especially as a disproportionate response to not receiving what they desire; discomfort with loud noises, experiencing soft touches as harsh and discomfort with rough food textures. Patients 4, 5, and 9 currently actively participate in sensory integration therapy. Table 1 presents a summary of each patient’s SI traits and how many patients experience each trait.

**TABLE 1:** Each patient’s sensory integration disorder trait and how many patients exhibited each trait according to surveys filled by caregivers.

Pt	Current age (yr)	Sex	*De novo* pathogenic variant	Tantrum outbursts	Discomfort with loud noises	Disproportionate experience of touch	Discomfort with rough food textures
**8**	7.5	M	c.13961A>G exon 43 (het) likely benign	5	4	4	3
**3**	5.5	M	c.14381A>G exon 46 (het) likely pathogenic	0	4	4	3
**7**	1.5	M	c.15142C>T exon 49 (het) likely pathogenic	4	5	3	5
**5**	3	M	c.15274T>C exon 49 (het) likely pathogenic	0	4	0	0
**2**	5.5	M	c.15397T>C p.Cys5133Arg exon 49 (het) likely pathogenic	5	0	3	0
**4**	8	M	c.15544G>C exon 49 (het) likely pathogenic	4	3	3	0
**9**	26	F	c.15641G>A exon 48 (het), pathogenic	3	5	2	0
**1**	12	F	c.16390A>C pT5434P (het) exon 52 likely pathogenic	3	4	3	0
**6**	3	M	c.6362C>A exon 32 (het) no data	3	5	3	2
				7/9	8/9	3/9	2/9

We noted the musical interest of three of our patients. They played a toy xylophone melodically, rhythmically tapped, and sang during the clinical visit. Patient 3 plays the piano and attends music therapy class, while patients 6 and 9 enjoy regularly listening to music and singing along. These patients tend to easily accurately recognize pitch.

All patients’ joint laxity was assessed subjectively upon physical examination and objectively according to the Beighton scale. In our group 6 out of 9 patients presented with a Beighton score indicative of joint hyperlaxity, and 3 out of 9 presented with a normal score. Assessment using the Beighton score is likely most reliable in children over the age of 6 years (Smits-Engelsman et al., 2011). Of the 4 patients older than 6 years in our group, two had a Beighton score indicative of joint hyperlaxity. All patients appeared to present with subjective joint hyperlaxity to varying degrees, especially while examining the joints of distal extremities.

Upon physical examination, patients presented with typical, yet mild, KS craniofacial features and dysmorphism. They all exhibited long palpebral fissures, sparse eyebrows, epicanthal folds, abnormal dentition, and high-arched palate (Figure 1). We observed astigmatism, cleft lip and palate, and heart and kidney disorders in our cohort, phenomena typical of Kabuki syndrome. Interestingly, patient 7 presented with coloboma (Figure 1).

Anthropometric measurements were analyzed using growth charts for patients with Kabuki Syndrome (Ruault et al., 2020) as well as World Health Organization (WHO) Growth Charts. Results are presented in Supplementary Table S1. According to the WHO Growth Charts, short stature can be observed in all patients in our cohort. Other anthropometric measurement results were deemed insignificant.

## 4 Discussion

We present a case series of ten patients who satisfy the criteria for a definitive Kabuki Syndrome diagnosis, with confirmed *KMT2D*-missense pathogenic variants—one with a confirmed variant in exon 39 and nine with confirmed variants located elsewhere, all near the C-terminus (Adam et al., 2019).

Data comparing and contrasting our group with *KMT2D* missense variants and those described in literature can be found in Supplementary Table S2. While analyzing this data from patients described in literature it can be seen that 80% presented with short stature and had at least one dysmorphism characteristic pertaining to Kabuki Syndrome, a finding that is consistent with our patient group (Bruni et al., 2021; Lehman et al., 2017). One-quarter of patients from literature presented with abnormal dentition or high-arched palate (6 out of 10 of our patients) (Cuvertino et al., 2020; Tharreau et al., 2022; Bruni et al., 2021; Badalato et al., 2017; Baldridge et al., 2020; Shangguan et al., 2019). Interestingly, seven patients described in the literature presented with brachydactyly, a finding that we did not observe in any of our patients (Cuvertino et al., 2020; Tharreau et al., 2022; Bruni et al., 2021; Badalato et al., 2017; Baldridge et al., 2020). Nine patients in literature presented with athelia or nipple hypoplasia—a finding that we observed only in our severely affected patient. Athelia was observed in patients with missense *KMT2D* pathogenic variants in any exon (Cuvertino et al., 2020; Tharreau et al., 2022; Baldridge et al., 2020). Moreover, upon analysis using the phenotyping application *Face2Gene* all of our patients with missense *KMT2D* variants had a moderately high or very high facial gestalt similarity to the typical KS phenotype.

**FIGURE 2:**
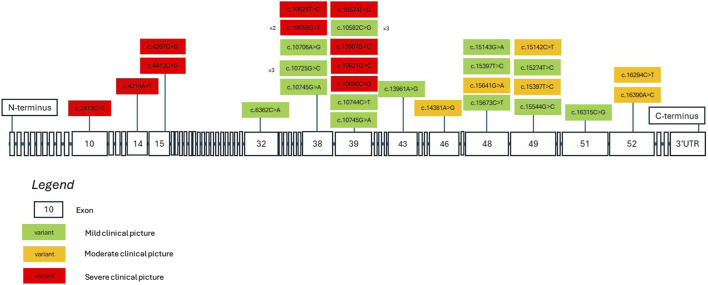
A *KMT2D* gene model showcasing missense variants identified in both our patients and in literature. The variants are color-coded to denote the severity of clinical presentations based on current clinical data. Severity is determined by specific criteria: moderate or severe developmental delay, drug-resistant seizures, moderate to severe vision or hearing impairment, certain heart defects, failure to thrive, premature death, and severe joint hyperlaxity. Patients with a severe clinical picture met more than one criterion, those with a moderate clinical picture met one criterion, and those with a mild clinical picture met none. Mild cases involved mild dysmorphism, minimal or no developmental delay, and characteristics that do not significantly hinder daily function.

**FIGURE 3:**
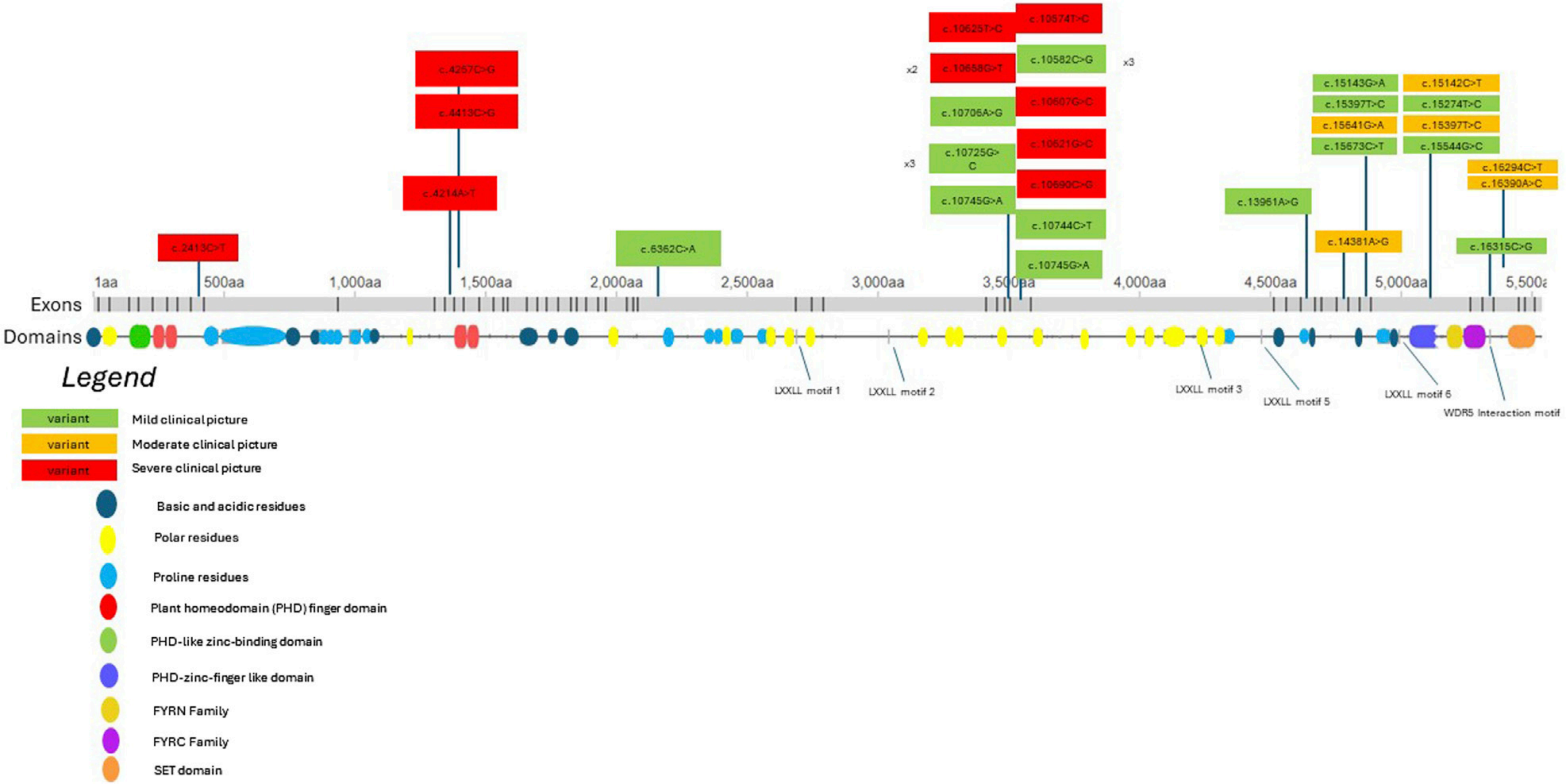
A KMT2D protein structure model showcasing different domains and the location of missense variants identified in both our patients and those described in literature. Clinical presentation severity is color-coded according to the legend in Figure 3. The abbreviation “aa” signifies “amino acids.” Domains are labeled according to the maps on *deciphergenomics.org* and *genecards.org*.

We aim to consolidate current knowledge regarding potential genotype-phenotype correlations linked to missense *KMT2D* variants. Figure 2 depicts a model of the *KMT2D* gene, illustrating both the missense variants identified in our patient group and those documented in existing literature. Variants are color-coded based on the severity of the associated clinical presentation, as determined by specific criteria: moderate or severe developmental delay, epilepsy refractory to treatment, moderate or severe vision or hearing impairment, certain heart defects, failure to thrive, premature death, and severe joint hyperlaxity (under 5 points according to the Beighton scale). Patients with a severe clinical picture met more than one criterion, those with a moderate clinical picture met one criterion and those with a mild clinical picture met none. Mild cases involved mild dysmorphism, minimal or no developmental delay and characteristics that do not significantly hinder daily function. The model suggests that missense variants located nearer to the N-terminus tend to be associated with a more severe clinical phenotype. Conversely, variants nearer to the C-terminus may manifest with a milder clinical phenotype. There are notable exceptions to this observation, indicating the necessity for cautious interpretation. Exons 38 to 39 show 7 out of 17 listed patient variants exhibiting a severe clinical picture. Building upon this, in order to better demonstrate which domains the listed variants may be influencing, Figure 3 depicts a model of the KMT2D protein structure. It links domains to the location of missense variants. It is noteworthy that LXXLL motifs 5 and 6 are essential for the association with the estrogen receptor alpha—the ESR1 nuclear receptor, and their mutation may crucially hinder the KMT2D protein function (Thul et al., 2017). No variant listed after LXXLL motifs 5 and 6 was associated with a severe clinical picture. Further research is imperative to validate and refine these findings.

Of the KS patients with missense variants described in the literature, 15 had congenital heart defects and one had epilepsy (Bruni et al., 2021; Lehman et al., 2017). None of our patients were diagnosed with epilepsy, and it may be a rarer finding in KS patients with missense pathogenic variants. Upon neurological examination it is noteworthy that all our patients presented abnormal posture (resulting from prominent kyphosis). Additionally, all patients presented with some degree of a kinematic stiffness pattern, a finding that may be associated with global hypotonia and kyphosis. Supplementary Table S2 comparing and contrasting our patients with those described in literature can be found in the Supplementary Material.

Of particular interest is the statistical analysis of neurodevelopmental characteristics to better nuance and demonstrate potential differences between patients with a missense pathogenic variant from our cohort as well as from literature with the phenotype of patients with a truncating variant (Adam and Hudgins, 2005). We chose to compare 5 neurodevelopmental milestones: sitting unsupported, walking unsupported, speaking 8 words, following simple commands, daytime toilet training. We compared the average age of milestone achievement in missense and truncating groups, and used Fisher’s Exact Test to determine a potential significant difference between the delays in the groups. There was no significant difference between the groups in all of the developmental milestones. Building on this finding, we aimed to search for a significant difference between the prevalence of joint hyperlaxity in our missense cohort and our 30 patients with truncating pathogenic variants using Fisher’s Exact Test. There was no significant difference between the groups. A study conducted in 2019 found the prevalence of joint hyperlaxity to be 31% in boys and 14% in girls with Kabuki Syndrome, with a total prevalence of 22% using the Beighton score above 3/9 points (Schott et al., 2019). In total out of 21 KS patients in our cohort in whom the Beighton score was assessed, 62% (13/21) presented with joint hyperlaxity. More details on our patients neurodevelopmental milestones can be found in Supplementary Table S3.

All of our patients presented with foot hyperesthesia; a finding not drawn attention to in literature. Namely, upon physical examination of the plantar reflex they expressed a noxious sensation followed by discomfort, a more severe condition than tickling. The children in our cohort who are unable to communicate verbally cried during this examination, and those able to communicate verbally expressed their discomfort. One explanation for this unusual behavioral pattern is sensory integration disorders (Kasdon and Fox, 2012). Upon surveyal, all caregivers in our group stated that their children had sensory integration disorder traits, while 3 patients currently actively participate in sensory integration therapy. These neurobehavioral abnormalities have been reported in KS patients, however foot hyperesthesia has never been described in KS patients in this context (Adam et al., 2011). We surveyed caretakers and asked them if and how often each of the selected four sensory integration disorder traits occurred on an integer scale from 0 to 5 (0 meaning the trait does not occur at all, 5 meaning the trait occurs several times daily). We also collected data from findings upon physical examination. Results are presented in Table 1. Using the two-way ANOVA statistical test we analyzed the impact of each of the traits by comparing the means of the different samples. The results determined a significant impact of the traits. This suggests that each sensory integration trait is dependent, i.e., if patients experience any one trait, they will likely experience another. Notwithstanding the above, this analysis has potential limitations. Data was quantified based on a qualitative survey and is thus subject to bias. Secondly our analysis is limited to four sensory integration traits. Thirdly the sensory integration traits themselves are subjective, namely, the excessiveness of tantrum outbursts, the loudness of noises, the degree of touch and the texture of food is subject to bias.

A second explanation for this behavioral pattern lies in the issue of brain plasticity. One study found that *KMT2D* haploinsufficiency in Kabuki Syndrome disrupts neuronal function through transcriptional and chromatin rewiring regardless of methylation. *KMT2D* deficiency disrupts neurogenesis, leading to a cell cycle disruption and precocious neuronal differentiation and ultimately altered neuronal plasticity. A reaction to this central nervous system development disruption could be altered plasticity as expressed by excessive sensations (Carosso et al., 2019).

Musicality in KS patients is only briefly described in literature (Schott et al., 2017). We observed a notable affinity for music among 3 individuals in our group. This finding brings to light a hitherto unexplored aspect of KS and prompts further investigation into the potential connections between this genetic disorder and musicality. Three of the patients actively played musical instruments and even sang during our examination, while one individual engages in piano playing as a hobby. Three participants are currently attending musical therapy. This anecdotal evidence suggests an association between Kabuki Syndrome and musical engagement. The factors contributing to this musical aptitude are not definitively established, but we can speculate on possible influences. Abnormal central nervous system plasticity leading to excessive sensation may also explain the tendency for musical inclination in KS patients. Care aimed at hearing development has already been used in KS patients with good results (Adam and Hudgins, 2005). Considering a potential tendency for heightened musical sensations in KS patients, it is possible that therapy aimed at developing musical skills may in turn improve central nervous system development and positively impact quality of life. Genetic predisposition, cognitive development, or sensory perception may play a role in fostering musical talents or interests among these individuals. However, it is essential to underscore that these suggestions remain speculative, and further research is needed to unravel the complex mechanisms underlying this phenomenon. The implications of these findings are wide-ranging. Musical engagement has the potential to positively impact the lives of individuals with Kabuki Syndrome, promoting cognitive, emotional, and social development. This information could inform therapeutic approaches and support strategies tailored to the unique needs of this patient population, recognizing the therapeutic value of music in their lives. We suggest that our findings be considered preliminary. Future research with larger and more diverse cohorts is warranted to corroborate and extend our observations, perhaps further investigating the effect of music or its therapy on KS patients’ quality of life.

Upon endocrinological examination, none of our patients presented with significant abnormalities. Neither hypothyroidism nor active hyperinsulinemic hypoglycemia were observed. Growth Hormone deficiency was not confirmed in any of our patients. KS is associated with growth hormone deficiency, autoimmune thyroiditis and rarely other endocrinological abnormalities (Nord, 2023). Only one of our patients presented with hypoglycemia during the newborn period, and during the second year of life with bleeding from her birth canal. This was most likely associated with a temporary increase in gonadotropin concentration and was treated as a self-limiting condition. No premature puberty characteristics were observed in the patients. When compared to literature (Supplementary Table S2), it can be noticed that 12 patients presented with chronic endocrinological problems, one with hypoglycemia and eleven with hypothyroidism. Due to a small number of patients and a wide variability in the findings, it is currently impossible to reach a conclusion regarding endocrinological manifestations in patients with Kabuki syndrome with confirmed missense pathogenic variants.

Missense pathogenic variants in our cohort occurred in SET (methylation) domains, FYR N-terminal domain and Zinc-Finger Proteins—transcriptional activators or repressors that bind DNA. Genotype-phenotype correlations among the variants falling within these two domains cannot be currently observed due to limited data. However, the biogenetic mechanism of disease-causing missense variants can be theorized. Missense variants in these domains may cause a disturbance in the *KMT2D* secondary structure through increased alpha-helical coiling, thus leading to impaired WRAD protein complex formation (Cocciadiferro et al., 2018; Cuvertino et al., 2020). Characteristic clustering of KS-associated variants in three protein domains was suggested, these are 1) germline variants associated with a KS phenotype; 2) variants found in cancer spectrum, and 3) control population missense variants observed in the ExAC database, not necessarily associated with specific phenotypes (Faundes et al., 2019). Animal models have suggested that *KMT2D* loss-of-function variants inhibit neural crest development. It is probable that, in the case of our patients with pathogenic variants occurring closer to the C-terminus, the ultimate WRAD protein complex formation was impaired to a lesser extent than it would have been in the case of a truncating pathogenic variant, thus leading to partial protein function and milder facial characteristics, for example, as seen in images of patients 7 and 8. On the other hand, it seems that despite the potentially milder clinical characteristics, their degree of developmental delay is not significantly different from that of patients with truncating variants, as seen by our data analysis. Moreover, a recent study aimed to characterize the molecular impact of *KMT2D* variants on the epigenetic and transcriptional landscapes of Kabuki Syndrome. It found that *KMT2D* exons 39 and 48 are particularly prone to mutations (up to one-third of KS variants occur in these exons) and additionally emphasized that exon 39 encodes long polyglutamine tracts which are crucial for protein function. This suggests the etiology of the rather severe clinical picture of our patient with a missense variant in *KMT2D* exon 39 (Jung et al., 2023).

This distinction between the phenotypic presentation in patients with missense pathogenic variants *versus* truncating pathogenic variants is concomitantly seen in other genetic syndromes. When examining patients with *NF1* missense variants, pulmonary artery stenosis and Noonan Syndrome clinical characteristics are commonly present as opposed to the neurofibromatosis clinical picture in patients with *NF1* truncating variants (Ben-Shachar et al., 2013). A whole yet functionally impaired protein most likely leads to heart disease (Ben-Shachar et al., 2013). Patients with *PTPN11* missense variants present clinically with Noonan Syndrome, whereas patients with *PTPN11* truncating variants present with metachondromatosis—a different clinical picture (Tartaglia et al., 2002). Truncating *KCNQ2* pathogenic variants frequently cause benign familial neonatal seizures, whereas missense pathogenic variants cause severe neonatal encephalopathy (Al Yazidi et al., 2017). These examples demonstrate that a different clinical picture can be seen in patients with missense variants as compared to those with truncating variants. Kabuki Syndrome patients with missense pathogenic variants close to the N-terminus often had confirmed functionally defective proteins and consequently presented with a phenotype like those patients with truncating pathogenic variants. In contrast, amino acid changes near the C-terminal domain of the protein tended to have minimal effects on H3K4 methylation, the *KMT2D* protein function (Cocciadiferro et al., 2018). This might explain the congenital malformation disorder phenotypic spectrum seen with KS patients with missense variants.

It is important to note that our relatively small patient cohort limits the degree with which conclusions may be reached. Much of our data collection required subjective and qualitative data analysis (as described in the sections above). While previous research on the topic can be found in literature, its relatively limited extent required a new research typology on our part. Therefore, further research aimed at analyzing exact locations of missense variants in KS patients and comparing them to the phenotype would help pinpoint personalized therapy, clinical targets for individual patients and contribute to the dissection of the once uniform phenotypic picture of chromatinopathies.

In conclusion our research refines the clinical and molecular picture of Kabuki Syndrome with a missense pathogenic variant etiology as compared to a truncating pathogenic variant etiology. KS patients with missense variants tend to have milder facial dysmorphism when compared to patients with truncating variants. They sometimes present with athelia, brachydactyly and different psychobehavioral disorders. The degree of their neurodevelopmental, psychosocial, and linguistic delay tends to be mild in certain cases, however not significantly different from thus far described KS patients with truncating pathogenic variants according to our data. These differences are suggested to stem from the nature and the location of the missense pathogenic variant (Cocciadiferro et al., 2018). Our research sheds light on similarities and differences in clinical picture between KS patients with missense pathogenic variants and those with truncating pathogenic variants.

## Data Availability

The datasets generated and analyzed for this study are available upon request to interested researchers.
