# Supplementary material for: Clinical and molecular characteristics of Kabuki syndrome patients with missense variants—novel features and literature review
Source: Front Genet. 2024 Jul 22;15:1402531. doi: 10.3389/fgene.2024.1402531 (PMC11298422; doi:10.3389/fgene.2024.1402531)
Supplement: Supplementary file 1 [file Table1.DOCX]

**Table S1 below:** Detailed clinical characteristics of 9 individuals with *KMT2D* missense variants outside of exons 38-39. Human Phenotype Ontology (HPO) for the terms presented are as follows: abnormal communication HP:0034434; abnormal dentition HP:0000164; abnormal emotionality HP:0100851; astigmatism HP:0000483; attention deficit HP:0007018; autistic behavior HP:0000729; cleft palate HP:0000175; coloboma HP:0000589; Dysarthria HP:0001260; epicanthal folds HP:0000286; foot hypersensitivity HP:5200058; gait imbalance HP:0002141; genu valgum; HP:0002857; high-arched palate HP:0000218; high-pitched voice HP:0001620; hyperreflexia HP:0001347; hypertelorism HP:0000316; hypotonia HP:0001252; impaired tongue muscle HP:0040173; kyphoscoliosis HP:0002751; long eyelashes HP:0000527; Long palpebral fissures HP:0000637; lower eyelid eversion HP:0007655; lumbar hyperlordosis HP:0002938; microcephaly HP:0000252; nystagmus HP:0000639; protruding ears HP:0000411; short stature HP:0004322; slanted palpebral fissures HP:0200006; sparse eyebrows HP:0045075; speech impediment HP:0002167; stereotypy HP:0000733; wide-based gait HP:0002136.

| Pt | Current Age (year) | Sex | De novo mutation | Prenatal and postnatal history | Significant medical problems | Dysmorphism | Physical assessment | Weight (kg), SD | Height (cm), SD | Head circum-ference (cm), SD | Sit | Walk | Follows commands | Speak 8 words | Toilet trained | School |
| --- | --- | --- | --- | --- | --- | --- | --- | --- | --- | --- | --- | --- | --- | --- | --- | --- |
| 8 | 7.5 | M | c.13961A>G, exon 43 (het),  ***ACMG:*** VUS, PM2, PP2. | G2, P2, CC, 39Hbd, 10 Apgar, birth weight 3700g, 50-85 WHO percentile | None | Long palpebral fissures, Sparse eyebrows, Epicanthal folds, Lower eyelid eversion, Abnormal dentition, High-arched palate | Kinematic stiffness pattern, genu valgum, nystagmus, normal hearing, hypotonia. Speech impediment, attention deficit. Foot hypersensitivity. Beighton 6 | 21.8,  0 SD | 116.0,  0 SD | 51.0  0 SD | 10month | 17month | 4year | 2.5year | 6year | Public |
| 3 | 5.5 | M | c.14381A>G exon 46 (het), ***ACMG:*** likely pathogenic, PS4, PM2, PP3, PP2. | G1, P1-preterm, 36Hbd, 8 Apgar, birth weight 2450g, 15-50 cc Integrative growth charts for preterm infants | Poor weight gain. cryptorchidism correction. | Slanted palpebral fissures, Long palpebral fissures, Long eyelashes, Lower eyelid eversion, Epicanthal folds, Hypertelorism, Sparse eyebrows, Protruding ears, Abnormal dentition, High-arched palate. | Stereotypy, speech impediment, abnormal communication - communicates with pictures-book tool. Lumbar hyperlordosis. Leans forward while walking. Stands up with straight legs. History of falling – gait imbalance. Astygmatism, trouble looking down (Cranial nerve-IV dysfunction susp.), Impaired motoric function of the tongue. Severe hypotonia, hyperreflexia. Foot hypersensitivity. Beighton 1. | 28.4,  +3 SD | 119.8,  +1 SD | 52.0  0SD | 1year | 24month | 1year | 2.5year | Does not | Integrative |
| 7 | 1.5 | M | c.15142C>T, exon 49 (het), ***ACMG:*** pathogenic, PS2, PM1, PP2, PM2, PM5, PP3 | G2, P2, term, no complications, birth weight 2750g, 3-15 WHO percentile | Congenital Pneumonia, Failure to thrive. | Coloboma, Lower eyelid eversion, High-arched palate, Protruding ears | High-pitched voice. Abnormal emotionality. Stereotypy. Wide-based gait, kyphoscoliosis. Short stature. Foot hypersensitivity. Beighton 9. | 7.2,  0 SD | 73.0,  -1 SD | 44.0  0 SD | 9month | 16month | 16month | Does not | Does not | Integrative |
| 5 | 3 | M | C.15274T>C, exon 49 (het), ***ACMG:*** likely pathogenic, PM2, PM5, PP3, PP2 | G3, P3-asphyxia, 37Hbd, 8 Apgar, birth weight 2850g, 3-15 WHO percentile | Congenital pneumonia. Cleft palate correction led to noticeably better hearing. Sensory integration therapy. | Slanted palpebral fissures, Long palpebral fissures, Long eyelashes, Lower eyelid eversion, Epicanthal folds, Sparse eyebrows, Protruding ears, Cleft palate, microcephaly. | Stereotypy, sudden stopping of psychomotor function - episodes of "losing focus" (seizure?). Speech impediment development, speaks in monosyllables. Thoracic hyperkyphosis. Impaired motoric function of the tongue. Hypotonia Hyperref. Foot hypersensitivity. Beighton 6. | 10.2,  0 SD | 86.5,  -1 SD | 45.5  0 SD | 16month |  | 1year | 1year | Does not | Integrative |
| 2 | 5.5 | M | c.15397T>C p.Cys5133Arg, exon 49 (het), ***ACMG:*** likely pathogenic, PS4, PM2, PP3, PP2 | G2, P2, NCB, 40Hbd, 10 Apgar, birth weight 2990g (15-50 WHO percentile) | Hypertension. Renal bivascularization. Thyroid nodules. feeding disorder – percutaneous endoscopic gastrostomy feeding. | Slanted palpebral fissures, Long palpebral fissures, Long eyelashes, Lower eyelid eversion, Epicanthal folds, Hypertelorism, Sparse eyebrows, Protruding ears, High-arched palate. | Dysarthria. Gait imbalance: knees bent while walking, wide gait. Thoracic hyperkyphosis. Hypotonia. Astygmatism. Left-ear hearing impairment. Foot hypersensitivity. Beighton 4. | 17.0,  0 SD | 108.4,  0 SD | 50.5  0 SD | 18month | 30month | 2.5year | 3.5year | 4year | School for people with special needs |
| 4 | 8 | M | c.15544G>C, exon 49 (het), ***ACMG:*** VUS, PM2, PP2, PP5 | G2, P2, 40Hbd, 10 Apgar, birth weight 4230g, 85-97 WHO percentile | Learning disability. Sensory integration therapy. | Slanted palpebral fissures, Long palpebral fissures, Long eyelashes, Lower eyelid eversion, Epicanthal folds, Hypertelorism, Sparse eyebrows, Protruding ears, Abnormal dentition | Gait imbalance. Stereotypy, also during sleep. Kinematic stiffness pattern. Severe genu valgum and foot position. Astygmatism. Severe Foot hypersensitivity (and whole body).  Beighton 9. | 27.2,  +1 SD | 130.2,  +2 SD | 51.5  0 SD | 9month | 18month | 3year | 1.5year | 4year | School for people with special needs |
| 9 | 26 | F | c.15641G>A, exon 48 (het), ***ACMG:***  pathogenic, PS2, PM2, PM5, PP3, PP2. | G2, P2, CC, 39Hbd, 9 Apgar, birth weight 3100g, 15-50 WHO percentile | Lymphatic edema, scoliosis, very mild KS symptoms. Sensory integration therapy. | Long palpebral fissures Sparse eyebrows, Epicanthal folds, lower eyelid eversion, abnormal dentition, high-arched palate. | Kyphoscoliosis, nystagmus. Abnormal emotionality – since teenage years very sensitive, perfectionist, autistic behavior. Thoracic hyperkyphosis. Beighton 2. | 49.8  -1SD | 162.0,  +1SD | 53.5  -2 SD | 11month | 12month | 1year | 1,5year | 2year | Public |
| 1 | 12 | F | c.16390A>C p.T5434P (het), exon 52, ***ACMG:*** likely pathogenic, PM2, PM5, PP3, PP2 | G2-multiple birth defects, P2, at term, 10 Apgar, birth weight 3200g (15-50 WHO percentile), hypoglycemia | Neonatal period: Pneumonia - respiratory support. feeding disorder. 2^nd^ year – birth canal bleeding. Cleft palate correction led to noticeably better hearing. | Slanted palpebral fissures Long palpebral fissures, Epicanthal folds, Lower eyelid eversion, Hypertelorism, Sparse eyebrows, Protruding ears, Abnormal dentition, Cleft palate surgical correction. | Speech impediment, hypotonia, mild gait imbalance, astygmatism, hyperreflexia. Complains of atypical pain, stereotypy. Foot hypersensitivity. Beighton 1. | 38.7,  0 SD | 131.4,  -1 SD | 53.0  0 SD | 1year | 26monthnth | 3year | 3.5year | 3year | Integrative |
| 6 | 3 | M | c.6362C>A, exon 32 (het), ***ACMG:*** VUS, PM2, PP2 | G3, P3, 39Hbd, 10 Apgar, birth weight 4020g, 85-97 WHO percentile | Toddler's diarrhea. Aortic coarctation treated surgically | Hypertelorism, near-lack of characteristic KS facial features. Short stature. | Kinematic stiffness pattern, yet normal for age. Psychosocial function appropriate for age. Speech impediment. Impaired motoric function of the tongue. Foot hypersensitivity. Beighton 6. | 13.2,  +0 SD | 91.2,  0 SD | 48.5  0 SD | 11monthnth | 20monthnth | 1year | 1.5year | Does not | Integrative |
