# Supplementary material for: Clinical and molecular characteristics of Kabuki syndrome patients with missense variants—novel features and literature review
Source: Front Genet. 2024 Jul 22;15:1402531. doi: 10.3389/fgene.2024.1402531 (PMC11298422; doi:10.3389/fgene.2024.1402531)
Supplement: Supplementary file 2 [file Table2.DOCX]

**Table S2 below:** Comparison and contrast between our patients with *KMT2D* missense variants and those described in literature. *This study does not distinguish between missense and other variants and reports data on all patients simultaneously. Human Phenotype Ontology (HPO) for the terms presented are as follows: abnormal dentition HP:0000164; athelia HP:0002561; Atrial Septal Defect (ASD) HP:0001631; brachydactyly HP:0001156; choanal atresia HP:0000453; cleft palate HP:0000175; Coarctation of Aorta (CoA) HP:0001680; Congenital Heart Disease (CHD) HP:0001627; Corpus callosum thinning HP:0033725; depressed nasal tip HP:0000437; developmental delay HP:0001263; epicanthal folds HP:0000286; Epilepsy HP:0001250; Eye abnormality HP:0000478; Hearing impairment HP:0000365; Heart Valve Abnormality HP:0001654; high-arched palate HP:0000218; hypertelorism HP:0000316; Hypoglycemia HP:0001943; Hypothyroidism HP:0000821; lacrimal gland anomaly HP:0011482; long eyelashes HP:0000527; Long palpebral fissures HP:0000637; lower eyelid eversion HP:0007655; microcephaly HP:0000252; micrognathia HP:0000347; neck pit HP:0100272; Patent Ductus Arteriosus(PDA) HP:0001643; Patent Foramen Ovale (PFO) HP:0001655; polydactyly HP:0010442; protruding ears HP:0000411; short stature HP:0004322; slanted palpebral fissures HP:0200006; sparse eyebrows HP:0045075; Strabismus HP:0000486; Ventricular Septal Defect (VSD) HP:0001629.

|  |  | Our study | Bruni et al., 2020 | Lehman et al., 2017* | Shanugguan et al., 2019 | Tharreau et al., 2022 | Badalato et al., 2017 | Baldridge et al., 2020 | Cuvertino et al., 2020 |
| --- | --- | --- | --- | --- | --- | --- | --- | --- | --- |
| Number of pts |  | 10 | 1 | 31 | 1 | 5 | 3 | 4 | 9 |
| Sex |  | M 8/10 | F | M 16/31 | F | M 2/5 | M 1/3 | M 1/4 |  |
| Age (years) |  | 1-12, 26 | 9 | 6 to 16 | 1 | Lifelong | 6,9,36 | 14-24 |  |
| Anthropometric measurements <3cc | Microcephaly | 1/10 | Yes | Yes | Yes |  |  | 4/4 |  |
|  | Short stature | 3/10 |  |  | No | 0 | 1/3 | 2/4 | 5/9 |
|  | Birth weight | Normal 10/10 | Yes |  |  |  |  |  |  |
|  | Birth length | Normal 10/10 | Yes |  |  |  |  |  |  |
| Facial dysmorphism | Typical KS features | 10/10 | Yes |  |  |  |  |  |  |
|  | Slanted palpebral fissures | 5/10 | Yes |  |  |  |  |  |  |
|  | Long palpebral fissures | 8/10 | Yes |  | Yes |  | 3/3 |  |  |
|  | Long eyelashes | 4/10 |  |  | No |  |  |  |  |
|  | Epicanthal folds | 7/10 | Yes |  | No |  |  |  |  |
|  | Hypertelorism | 6/10 | Yes |  | No |  |  |  |  |
|  | Sparse eyebrows | 7/10 | Yes |  | No |  |  |  |  |
|  | Protruding ears | 5/10 | Yes |  | Yes | 0 | 0/3 | 3/4 | 6/9 |
|  | Micrognathia | 1/10 | Yes |  | No |  |  |  |  |
|  | Lower eyelid eversion | 9/10 |  |  | No |  |  |  |  |
|  | Depressed  nasal tip | 9/10 |  |  | Yes |  |  |  |  |
|  | Choanal atresia | 1/10 |  |  |  | 1/5 | 3/3 | 4/4 | 7/9 |
|  | Lacrimal gland anomaly | 1/10 |  |  |  | 3/5 | 0/3 | 2/4 | 7/9 |
|  | Neck pits | 0/10 |  |  |  | 2/5 | 1/3 | 1/4 | 7/9 |
| Mouth dysmorphism | Abnormal dentition | 10/10 | Yes |  | No | 1/5 | 3/3 | 4/4 | 2/9 |
|  | Macrodontia |  | Yes |  |  |  |  |  |  |
|  | High-arched palate | 6/10 | Yes |  | Yes |  | 1/3 |  |  |
|  | Cleft palate | 2/10 | No | 5/26 |  |  |  |  |  |
| Finger dysmorphism | Brachydactyly | 0/10 | Yes |  |  | 1/5 | 2/3 | 1/4 | 2/9 |
|  | Polydactyly | 0/10 |  |  |  | 1/5 |  |  |  |
| Prenatal history |  |  |  |  |  |  |  |  |  |
| Other dysmorphism | Athelia | 1/10 |  |  |  | 1/5 | 0/3 | 2/4 | 6/9 |
| Cardiovascular | CHD | 5/10 as follows: | No | 10/26 |  | 0/5 | 1/3 | 0/4 | 3/9 |
|  | CoA | 1/10 |  |  | Yes | 0/5 |  |  |  |
|  | ASD | 0 |  |  |  | 0/5 | 1/3 |  |  |
|  | VSD | 1/10 |  |  |  | 0/5 |  |  |  |
|  | PDA | 1/10 |  |  |  | 0/5 |  |  |  |
|  | PFO |  |  |  | Yes | 0/5 |  |  |  |
|  | Heart valve abnormality | 2/10 |  |  |  |  |  |  |  |
| Respiratory |  | 0 | No |  |  | 0/5 | 0/3 | 1/4 | 2/9 |
| Neurological | Epilepsy | 0 | Yes |  | No |  |  |  |  |
|  | Developmental delay | 10/10 | Yes |  | No |  | 3/3 |  |  |
|  | Corpus callosum thinning | 0 |  |  | No | 0/5 | 0/3 | 2/4 | 0/9 |
|  | Eye anomaly | 0 |  | 10/21 |  | 0/5 |  |  |  |
|  | Strabismus | 0 |  |  | No |  |  |  |  |
|  | Hearing impaired | 4/10 |  | 5/22 | Yes | 4/5 | 3/3 | 4/4 | 8/9 |
|  |  |  |  |  |  |  |  |  |  |
| Endocrine | Hypoglycemia | 1/10 |  |  | Yes |  |  |  |  |
|  | Hypothyroidism | 0/10 |  |  |  | 1/5 | 0/3 | 4/4 | 6/9 |
| Genetics | Missense variant | c.16390A>C, c.15397T>C, c.14381A>G, c.15544G>C, C.15274T>C, c.6362C>A, c.15142C>T, c.13961A>G, c.15641G>A, c.10607G>C | c.2413C>T | 8/31  c.4214A>T  c.4413C>G  c.4267C>G  c.15673C>T  c.15397T>C  c.15143G>A  c.15142C>T  c.16315C>G | c.16294C>T | c.10706A>G, c.10745G>A | c.10725G>C all pts | c.10574T>C, c.10582C>G, c.10621G>C, c.10690C>G | c.10582C>G, c.10582C>G, c.10625T>C, c.10658G>T, c.10658G>T, c.10745G>A, c.10744C>T |
|  | Exon | 52, 49, 46, 49, 49, 32, 49, 43, 48, 39 | 10 | 14, 15, 15, 48, 48, 48, 48, 51 | 52 | 38, 39 | 38 | 39, 39, 39, 39 | 38, 38, 38, 38, 38, 38, 39 |
