# Supplementary material for: Clinical and molecular characteristics of Kabuki syndrome patients with missense variants—novel features and literature review
Source: Front Genet. 2024 Jul 22;15:1402531. doi: 10.3389/fgene.2024.1402531 (PMC11298422; doi:10.3389/fgene.2024.1402531)
Supplement: Supplementary file 3 [file Table3.DOCX]

**Table S3:** Ages of selected developmental milestone achievement.

| Pt | Current Age (yr) | Sex | De novo mutation | Sit support | Sit no support | Walk by self | Pincer grasp | Uses spoon | Self drinks from bottle | Eats solid food by self | First word | 8word vocab | Simple phrase | Reacts to own name | Follows command | Counts to 10 | Understands time | Toilet trained |
| --- | --- | --- | --- | --- | --- | --- | --- | --- | --- | --- | --- | --- | --- | --- | --- | --- | --- | --- |
| 8 | 7.5 | M | c.13961A>G, exon 43 (het), likely benign | 7months | 10 months | 17months | 12months | 18months | 24months | 24months | 3years | 2.5years | 4years | 3years | 4years | Does not | 6years | 6years |
| 3 | 5.5 | M | c.14381A>G exon 46 (het), likely pathogenic | 10months | 13 months | 24months | Does not | 36months | 30months | 30months | 19months | 2.5years | 3.5years | 1years | 1years | 3.5years | 3years 4months | No data |
| 7 | 1.5 | M | c.15142C>T, exon 49 (het), likely pathogenic | 7months | 9 months | No data | 7months | 11months | 8months | 12months | 7years | No data | Does not | Does not | No data | Does not | Does not | No data |
| 5 | 3 | M | C.15274T>C, exon 49 (het), likely pathogenic | No data | 16 months | No data | No data | 22months | No data | No data | 15months | 10months | Does not | No data | 10months | No data | No data | No data |
| 2 | 5.5 | M | c.15397T>C p.Cys5133Arg, exon 49 (het), likely pathogenic | 15months | 18 months | 30months | 24months | 39months | 45months | Does not | 2.5years | 3years 4months | Does not | 2years | 2years 3months | Does not | Does not | 3years 10months |
| 4 | 8 | M | c.15544G>C, exon 49 (het), likely pathogenic | 7months | 9 months | 18months | 5months | 25months | 27months | 30months | 14months | 1.5years | 20months | 1.5years | 3years | 5.5years | 5years | 4years |
| 9 | 26 | F | c.15641G>A, exon 48 (het), pathogenic | 9months | 10 months | 24months | No data | 22months | Does not | Does not | 23months | 2years | Does not | 11months | 1.5years | Does not | Does not | No data |
| 1 | 12 | F | c.16390A>C .p T5434P(het)ekson 52, likely pathogenic | 12months | 20 months | 26months | Does not | 40months | 48months | 84months | 3years | 3.5years | 3years 9months | 1years | 3years | 8years | 8years | 3years |
| 6 | 3 | M | c.6362C>A, exon 32 (het), no data | 11months | 12months | 20months | 7months | 12months | 12months | 12months | 1.5years | 1.5years | 2years 2months | 8months | 1years | Does not | 1years 4months | No data |
